# The Calm after the Storm: A State-of-the-Art Review about Recommendations Put Forward during the COVID-19 Pandemic to Improve Chronic Pain Management

**DOI:** 10.3390/jcm12237233

**Published:** 2023-11-22

**Authors:** Marimée Godbout-Parent, Tristan Spilak, M. Gabrielle Pagé, Manon Choinière, Lise Dassieu, Gwenaelle De Clifford-Faugère, Anaïs Lacasse

**Affiliations:** 1 Département des Sciences de la Santé, Université du Québec en Abitibi-Témiscamingue (UQAT), Rouyn-Noranda, QC J9X 5E4, Canada; marimee.godbout-parent@uqat.ca (M.G.-P.);; 2 Centre de Recherche, Centre Hospitalier de l’Université de Montréal (CHUM), Montréal, QC H2X 0A9, Canada; 3 Département D’anesthésiologie et de Médecine de la Douleur, Faculté de Médecine, Université de Montréal, Montréal, QC H3T 1J4, Canada

**Keywords:** chronic pain, COVID-19, pandemic, management, care treatment, recommendations, solutions, review

## Abstract

The COVID-19 pandemic has brought its fair share of consequences. To control the transmission of the virus, several public health restrictions were put in place. While these restrictions had beneficial effects on transmission, they added to the pre-existing physical, psychosocial, and financial burdens associated with chronic pain, and made existing treatment gaps, challenges, and inequities worse. However, it also prompted researchers and clinicians to seek out possible solutions and expedite their implementation. This state-of-the-art review focuses on the concrete recommendations issued during the COVID-19 pandemic to improve the health and maintain the care of people living with chronic pain. The search strategy included a combination of chronic pain and pandemic-related terms. Four databases (Medline, PsycINFO, CINAHL, and PubMed) were searched, and records were assessed for eligibility. Original studies, reviews, editorials, and guidelines published in French or in English in peer-reviewed journals or by recognized pain organizations were considered for inclusion. A total of 119 articles were analyzed, and over 250 recommendations were extracted and classified into 12 subcategories: change in clinical practice, change in policy, continuity of care, research avenues to explore, group virtual care, health communications/education, individual virtual care, infection control, lifestyle, non-pharmacological treatments, pharmacological treatments, and social considerations. Recommendations highlight the importance of involving various healthcare professionals to prevent mental health burden and emergency overload and emphasize the recognition of chronic pain. The pandemic disrupted chronic pain management in an already-fragile ecosystem, presenting a unique opportunity for understanding ongoing challenges and identifying innovative solutions. Numerous recommendations were identified that are relevant well beyond the COVID-19 crisis.

## 1. Introduction

The global population have seen their health and lives affected by the COVID-19 pandemic and its numerous consequences [1]. In fact, the pandemic has caused a combination of physical consequences (e.g., virus-related dry cough, fever, respiratory difficulties, fatigue [2], long-haul COVID [3]), psychosocial consequences (e.g., psychological distress, limited access to health services, domestic violence [4]), and economic consequences (e.g., business closures, increased unemployment rates, reduced work hours [5]). During the crisis, efforts were rapidly deployed to help people affected by the virus and to control its spread as much as possible [6]. The COVID-19 pandemic and its consequences mentioned above have disproportionately affected vulnerable groups, such as persons marginalized by their social identities, the elderly, people living with disabilities, women, and people with chronic illness [4,7]. For example, the pandemic has exacerbated the physical, psychological, economical, and health challenges that people living with chronic pain (CP) face on a daily basis [8,9,10]. Even before the COVID-19 pandemic, CP was an under-reported, under-recognized, under-diagnosed, and frequently under-treated disease [11,12,13,14]. Several barriers are named as potential sources leading to this suboptimal management, such as the lack of access to multidisciplinary care and the suboptimal integration of multimodal approaches which seek a balance between pharmacological and non-pharmacological treatments [13,15,16,17]. The restrictions imposed by public health during the COVID-19 pandemic, including lockdowns, the closure of non-essential services, and requirements to stop in-person treatment, have affected the accessibility of treatment, therefore potentially causing significant harm to people living with CP [8,9]. The pandemic has also worsened pre-existing physical, psychological, and financial burdens associated with CP and increased risk factors such as reduced sleep, inactivity, fear, anxiety, and depression [10,18]. 

COVID-19 has certainly intensified the existing gaps, difficulties, and inequalities in treatment for people living with CP, but has also emphasized the magnitude of the disease and created a sense of urgency in research [8]. In the most urgent time of the pandemic, much research was conducted on the impacts of the pandemic and many recommendations were made by researchers, experts, and healthcare professionals. Considering the current slower COVID-19 transmission rates and the concomitantly ongoing recovery of our healthcare systems from such a trial, it is vital to analyze the research carried out during this period. This is relevant not only to prepare for potential new pandemics, but to harness recommendations issued during the crisis that could help improve pain management well beyond the COVID-19 crisis. Therefore, this study represents a state-of-the-art review, conducted to synthesize concrete recommendations issued during the COVID-19 pandemic (2019–2021) for improving the health and maintaining the care of people living with CP.

## 2. Methodology 

A “state-of-the-art” review [19] was conducted to address the state of knowledge regarding suggested improvements for the management of CP during the first 20 months of the COVID-19 pandemic and to classify the recommendations to be implemented. This type of review is time-bound in terms of literature temporal exhaustiveness and focuses on rapidly but methodically searching the current literature to address contemporary issues; its results focus on knowledge and priorities for future investigation and research [19].

***Eligibility criteria.*** This review considered original studies, reviews, editorials, and guidelines that have been published in peer-reviewed journals or in some reports/statements issues by recognized pain organizations (e.g., International Association for the Study of Pain, Canadian Pain Society), or other grey literature. The articles had to focus on adults (age ≥ 18 years old) living with CP (pain that persists for more than 3 months [20]) of non-cancerous origin. They also had to be published in English or in French between December 2019 and July 2021. As an example, this period corresponded to the first three COVID-19 waves of the pandemic in Canada [21]. The present review thus allowed us to harness the recommendations published during the crisis and to put them into perspective with the current situation in healthcare facilities that have slowly recovered from the COVID-19 crisis. 

***Exclusion criteria.*** Preclinical studies were excluded, as well as articles specifically addressing molecular aspects of pain, post-COVID syndrome (long COVID), cancer pain or pediatric pain.

***Information sources and search strategy.*** Studies were retrieved on 1 July 2021 by searching the following computerized databases: Medline (Ovid), PsycINFO (Ovid), CINAHL (EBSCOhost), and PubMed (past 7 days). The search on PubMed was made to capture potential new articles indexed on PubMed but not yet on Ovid (as both are windows for Medline but PubMed indexation is sometimes more rapid) [22].

The search strategy was developed in collaboration with an experienced medical librarian of the *Centre Hospitalier de l’Université de Montréal* (CHUM). The strategies were peer-reviewed by another senior information specialist prior to executing using the PRESS Checklist [23]. The search strategy included synonyms for: (1) CP and (2) COVID-19 (File S1). Different types of chronic non-cancer pain conditions (e.g., CP in general, neuropathic pain, fibromyalgia, arthritis, back pain, and migraine) as defined by the International Association for the Study of Pain (IASP) Task Force for the Classification of Chronic Pain [24] were included in the search strategy. 

***Study selection.*** All citations were entered in the citation management software Endnote X9^®^ and duplicates were removed by the librarian using the method reported by W. Bramer [25]. A fast process was favoured, so the selection process was achieved by one trained reviewer with medical expertise (TS) rather than two. Firstly, titles and abstracts were screened according to the inclusion criteria. Secondly, full texts of previously selected studies were reviewed to assess their eligibility.

***Data Extraction.*** The data collection process was carried out using a standardized extraction form that was pretested and improved with a sample of 14 studies at the beginning of the extraction process. For each study meeting the eligibility criteria, the following information was retrieved: date of publication, authors, country of data collection (if applicable), the type of article, and concrete recommendations to improve health and maintain care for people living with CP. In order to remain as precise as possible, the recommendations have been extracted keeping the authors’ wording. Relevant data were also extracted to allow the classification of recommendations and to check whether they were made for specific populations (e.g., elderly, migraine populations, fibromyalgia, specific cultural groups, etc.). All this information was collected in an Excel^®^ spreadsheet. 

***Synthesis of results.*** The various recommendations retrieved from studies were described, combined in tables, and classified into 12 categories. The categories were chosen by consensus after the analysis of all the recommendations issued. When a recommendation could be inserted in more than one category, a choice was made by two of the authors (MG-P and AL) on the most representative category.

## 3. Results

**Figure 1:**
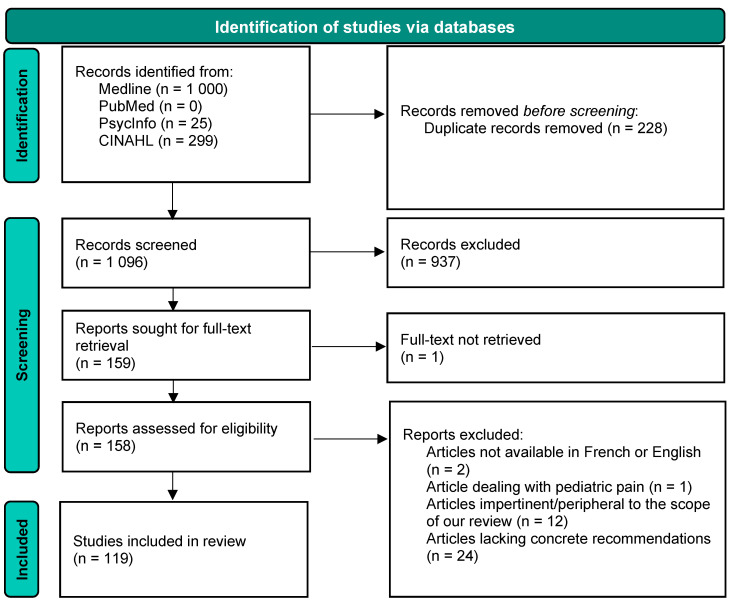
Study selection flow diagram.

**Figure 2:**
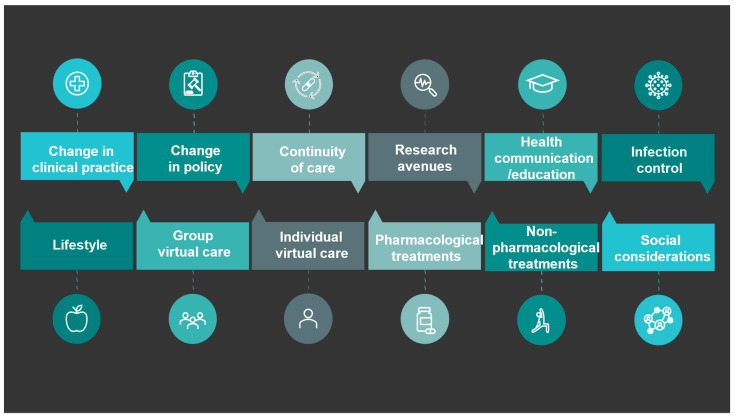
Categories of recommendations.

Flow diagram representing the study selection process is shown in Figure 1. After assessing the articles for eligibility, a total of 119 articles were included in the study. The characteristics of these studies and their respective recommendations to improve health and maintain care for people living with CP are detailed in File S2. From the 119 articles, over 250 concrete recommendations were extracted and then reduced to 150 recommendations to minimize redundancies. Recommendations were then classified into 12 distinct categories: change in clinical practice, change in policy, continuity of care, research avenues to explore, group virtual care, health communication/education, individual virtual care, infection control, lifestyle, non-pharmacological treatments, pharmacological treatments, and social considerations (Figure 2). For the sake of the brevity of this report, two illustrative recommendations from each category were selected by the research team and are presented in Table 1. The complete list of recommendations is presented in File S3.

## 4. Discussion

This state-of-the-art review resulted in 12 categories of concrete recommendations (evidence-based or from different experts) issued during the COVID-19 pandemic. The recommendations consider many areas of healthcare that must be taken into consideration in order to hope for positive changes in CP management. By focusing on the recommendations issued in crisis time, this article provides an opportunity to describe and classify solutions that have been issued to improve the health and maintain the care of people living with CP well beyond the pandemic.

### 4.1. Virtual Care

The literature underlined that restrictive measures put in place by the government, along with the beginning of the lockdown, created new uncertainties regarding the continuity of healthcare services for the treatment of CP [9]. Moreover, difficulties accessing medical services, medication refills, and non-pharmacological treatments have been reported [8]. Knowing the importance of a multimodal approach for the management of CP, accessibility to non-pharmacological care was a major issue during the pandemic [8]. To address this gap, recommendations were made to provide the opportunity to offer non-pharmacological treatments in a virtual manner. The use of synchronous videoconferencing to deliver pain management programs [38] and the implementation of virtual support groups have been recommended [39]. These group meetings allowed patients to break the isolation brought on by the pandemic [39]. Indeed, some have reported that online support groups played an important role in their psychological well-being during the pandemic [9]. In addition to virtual treatment, communication and education can also be provided through virtual modalities. The development of online pain acceptance programs or self-management programs (e.g., *Agir pour moi* program [56]) could not only help patients, but also clinicians and patients’ families [41]. Healthcare professionals and support groups’ organizations could also use online modalities for interventions, and knowledge transfer activities regarding alternative physical/psychological approaches, allowing CP patients to still benefit from these kinds of treatment [29]. For example, education on possible exercises to do at home to stay active despite the confinement, meditation exercises, or relaxation techniques could be provided via online modalities, and were recommended as important elements to ensure the continuity of care [47,48]. Despite the importance of increasing the accessibility of virtual care [53,54], studies should be conducted to validate the effectiveness of virtually conducted treatments [37], and special attention should be paid to inequalities in access to care. Indeed, although virtual care can be beneficial for persons living outside of large urban centres, it can be more challenging for individuals with low digital literacy (e.g., members of the elderly population) or people living in isolated geographical areas (i.e., with limited access to the internet). Also, a communication of trust is more difficult to establish with healthcare professionals [9,57]. One Canadian study reported that nearly 15% of patients were not well equipped to receive virtual care [58]. Despite some accessibility challenges, the development of effective non-pharmacological treatments delivered virtually may be essential during any future pandemic, especially when lockdowns are in place. Many multidisciplinary pain treatment facilities in Canada are now well prepared to deliver virtual care and consider virtual care to be sustainable for any future pandemic and well beyond [58]. It is also a good opportunity for healthcare professionals to reinforce the utility of effective non-pharmacological treatment options and their benefits in pain management [29]. The COVID-19 pandemic developments have accelerated the adoption of virtual care and have brought huge benefits (e.g., cost saving for patients [59], improved access and efficiency [60], and greater geographical reach for clinicians [58]), even while still being a work in progress [61].

### 4.2. Involvement of Different Healthcare Professionals and Mental Health Burden

Long before the pandemic, a multidisciplinary approach was recommended for CP management [15]. As this condition is responsible for many physical, psychological, and emotional consequences, many key players must be involved in order to ensure adequate management (e.g., psychologists, physicians, nurses, pharmacists, and physiotherapists) [11]. The pandemic brought a climate of fear and a considerable increase in the patients’ level of stress, anxiety, and depression [62,63]. Considering that the impacts on Considering that the impacts on physical health, and mental health, and well-being may be heightened during times of stress periods [10], it is now clear that the pandemic will have increased psychological distress in the general population [64,65] and people living with CP [9]. Indeed, in a study on a population of people living with rheumatoid arthritis, it was reported that the lockdown had increased pain and impairment of function, both of which were linked to increased rates of depression, anxiety, low self-esteem, insomnia, and other mental health problems [26]. In the light of these observations, it was recommended that the participation of psychiatrists and psychologists in CP management be increased in order to deal with this incoming surge of mental illnesses [26]. Even though psychologists are often involved in tertiary care multidisciplinary teams [66], different barriers prevent patients from being able to consult these specialists for their pain management (e.g., access is limited and patients end up having to consult privately, which can directly cause a financial limit [8], and there is a shortage of trained pain psychologists [67]). Other allied healthcare professionals such as social workers should thus be involved. Since interdisciplinary interventions where staff work together can minimize psychological distress [55], it is, therefore, important that policymakers prevent the pandemic’s harmful effects on the mental health of people living with CP and make accessible psychosocial interventions rapidly [9]. Furthermore, accessible psychosocial interventions that take into consideration the most socially and economically vulnerable are required [9], and are recommended to deal with the impacts of the pandemic. Recognizing that mental health challenges existed in people with CP before the pandemic, and that COVID-19-related anxiety may persist, [68] a multidisciplinary approach with psychologists remains relevant beyond the pandemic.

### 4.3. Involvement of Different Healthcare Professionals and Overcrowded Emergency Departments

In addition to increasing skills to deal with the negative consequences that the pandemic had on people living with CP, collaboration between healthcare professionals could help to decrease the number of patients with CP who attend already-overcrowded emergency departments. This problem was known long before the COVID-19 pandemic, but was exacerbated during this period [69]. The emergency department is usually not the appropriate place to address the complex needs of CP patients where physical, cognitive, behavioural, and psychosocial assessments are required for the comprehensive management of CP [70,71]. The collaboration between multidisciplinary pain treatment facilities and community/primary care healthcare teams seems to be part of the solution [27], and can include preparing “rescue care” plans (timely and appropriate measures to stabilize a patient’s condition during emergencies), the home delivery of medications, and self-administered therapies [27,28]. In the past, community healthcare teams have effectively supported efforts to manage epidemics, including the H1N1 and Ebola epidemics [72,73]. Implementing community/primary-care multidisciplinary healthcare teams is important not just during the pandemic, but beyond it as well.

### 4.4. Recognition of CP and Its Consequences

Despite the many consequences associated with CP, this condition remains stigmatized and under-recognized in clinical practice and in the general population [11,12]. Even before the pandemic, people living with CP already faced many challenges in obtaining a diagnosis and being believed to be legitimate patients by healthcare professionals [74,75,76,77,78]. As the pandemic exacerbated challenges arising from this under-recognition, several new recommendations have been issued to move towards a greater recognition and acceptance of CP. Indeed, the need for an improved recognition of CP and its associated physical limitations has been highlighted, and such an improvement can be achieved by prioritizing continued education and advocacy amongst the public, healthcare professionals, and policymakers. Such advocacy could, for example, push for the provision of material compensation for pain-related disabilities [9] (e.g., assistive devices, transportation, universal insurance coverage for non-pharmacological approaches and services). In the same way, there is a need for CP to be treated as an urgent condition and for healthcare professionals to feel morally and ethically obliged to offer adequate treatments, and to ensure continued support for these patients [32,34]. A better recognition of CP will help advance research, understand the true magnitude of the problem, and consequently improve the chances of patients receiving viable treatment options [79]. In addition, there is a need for a greater awareness, identification, and reduction in conscious and unconscious biases that can affect the care of CP patients from vulnerable and/or minority groups [9,55]. These numerous recommendations echo the work of various large-scale working groups such as Health Canada’s Canadian Pain Task Force [13,14,80]. While there is still a long way to go to overcome the under-recognition and stigma surrounding the disease, the recent work and recommendations of the Canadian Pain Task Force and the present review may certainly help move things forward.

Whether through virtual care or the better integration of key healthcare professionals in chronic pain management, several recommendations emerged during the COVID-19 pandemic. The improved management of CP can be hoped only if the stigma decreases and it becomes better recognized.

### 4.5. Strengths and Limitations

Despite the advantages of a “state-of-the-art review” (e.g., rapid but comprehensive searching of the current literature to state the knowledge and address recommendations or future investigations needed [19]), this type of review does not take into account the quality of the studies from which the data was extracted. For feasibility purposes, only some electronic databases were used in this study (Medline, PsycINFO, CINAHL, and PubMed), which may have led to potential articles being missed. The strength of the recommendations can thus not be assessed. Also, although we conducted the review between December 2019 and July 2021 (during the first three COVID-19 waves in Canada), some information could have been published thereafter and other recommendations may have been missed. This temporal cut-off may have influenced the results. Nevertheless, the present review allows us to harness the recommendations published during the crisis and put them into perspective with the current situation in healthcare facilities that have slowly recovered from the crisis. Most recommendations were formulated at the outset of the pandemic, and we are now better prepared to adapt our healthcare practices and methods of delivering care for CP patients in future crises. 

## 5. Conclusions

The COVID-19 pandemic has undoubtedly exacerbated the existing physical, psychological, and economic burden associated with CP. However, it has also provided a unique opportunity for all stakeholders involved in CP management to collaborate and swiftly devise solutions for better care. Numerous recommendations regarding treatments, clinical practice, policy, and research avenues have been proposed to address existing pain management deficiencies. The pandemic has showcased the potential and effectiveness of virtual care, contributing to its wider acceptance as a treatment method. Critical aspects that have often been overlooked, such as the mental health of individuals living with CP, were reinforced. The ongoing battle to provide equitable, effective treatment and combat stereotypes surrounding CP is far from being over, but the numerous insights shared by the scientific community reinforce awareness and propel us in a promising direction. As the pandemic’s hold weakens, it is crucial to make use of the research carried out during this crisis and incorporate it into our healthcare system. 

This literature synthesis summarizes recommendations to better prepare for future pandemics and extends our knowledge well beyond the confines of this particular crisis. Future research could be conducted to verify whether the recommendations issued between 2019 and 2021 are applicable to or implemented in the current healthcare system.

## Figures and Tables

**Table 1:** Illustrative recommendations for the 12 different categories.

Recommendation Categories	Recommendations
**Change in clinical practice**	Need for increased participation of psychiatrists and psychologists to support psychological distress [26]. Need for a reinforcement of non-urgent healthcare services and outpatient assistance to redirect CP patients away from overly saturated emergency services in an environment of scarce resources, which can be achieved by means of increased collaboration between multidisciplinary pain treatment facilities and community/primary care healthcare teams. This can include preparing “rescue care” plans, home delivery of medications, and self-administered therapies to keep patients away from emergency departments and limit their exposure to COVID-19 [27,28].
**Change in policy**	Need to improve the recognition of CP experience and related physical limitations, through information and advocacy to the public, healthcare professionals, and policymakers [9]. Urgency that policymakers take action to prevent the pandemic’s detrimental effects on mental health of people living with CP, especially the most socially and economically vulnerable. Implementing accessible psychosocial interventions tailored to these vulnerable populations appears essential [9].
**Continuity of care**	Need for routine follow-up visits, whether conducted virtually (telehealth options) or in person. These visits can be performed by any member of the healthcare team and should be time-limited, focused, and regularly scheduled. These encounters will allow for assessing current symptoms, adherence to medication regimens, and the presence of any red-flag concerns [29,30,31]. Need for CP to be treated as an urgent condition and for healthcare professionals to feel morally and ethically obliged to ensure continued support for CP patients. Some may require urgent pain consultations, interventions, or medication titration and refills, as the deferment of multidisciplinary pain treatment facilities consultations and limited access to primary care can increase morbidity and mortality [32,33,34].
**Research avenues to explore**	Need for studies evaluating the long-term impact of the pandemic on pain-related, emotional, and psychological outcomes in CP patients, and determinants implicated in such outcomes (e.g., coping skills, self-management) [35,36]. Need for studies evaluating the efficacy of non-pharmacological treatment modalities conducted virtually [37].
**Group virtual care**	Evaluate whether the use of synchronous videoconferencing to deliver pain management programs is worthwhile, and if so, how to develop, deliver, and measure outcomes of such programs [38]. Implementation of group virtual care to benefit patients struggling with loneliness and social isolation, in addition to helping address concerns in overwhelmed clinicians [39].
**Health communication/education**	Need to ensure that key information regarding serious infectious diseases is as accurate and transparent as possible with timely updates. Public administration and media should facilitate public awareness and reduce false rumours that can cause widespread panic and unease [40]. Provide online resources that can disseminate pain education and online self-management programs that can be developed for those living with pain, those close to them, and healthcare professionals [41].
**Individual virtual care**	Healthcare professionals and support group organizations should implement individual virtual care options to supplement safe in-person care. Online interventions and knowledge transfer activities could also prioritize informing and empowering people living with CP regarding alternative physical/psychological approaches when the usual ones are not feasible. Harnessing the web could enhance treatment access for people living with CP well beyond the COVID-19 pandemic [8]. Mobile health apps for headache documentation such as Migraine Buddy, Migraine Coach, and Migraine Monitor have been shown to be useful in improving communication between patients and physicians. A balance between the amount of data collected by the app for clinical purposes and the patient’s perception of satisfaction must be assessed. Likewise, physicians should allow for an adjustment period when introducing an app to their patients and ensure that they have been well informed on how to use it [42].
**Infection control**	Triage of pain patients may be helpful in terms of differentiating those who may be adequately treated by virtual care versus in-clinic consultations. Triage factors may also include acuity and severity of pain, whether or not the patient has comorbid psychiatric conditions, but also occupational risks of infection (e.g., whether the patient is a first responder) and social situation (e.g., whether the patient is also a caregiver or has children) [43,44,45]. Reinforcing patients’ and healthcare workers’ education on fundamental preventive gestures, such as frequent hand washing, social distancing, and symptom monitoring will aid in reducing transmission of infectious diseases [46].
**Lifestyle**	Need for healthcare professionals to provide patients with proper education and guidance on ways to stay physically active at home during future pandemics [47,48]. Migraine patients could benefit from lifestyle changes associated with intelligent lockdown, such as working from home, scaling down demanding social lives, and freedom to choose how to organize one’s time [49].
**Non-pharmacological treatments**	Healthcare professionals should take the opportunity to further reinforce the utility of effective nonpharmacologic treatment options including graded exercise, healthy lifestyle, meditation, and meditative movement activities (tai chi and yoga), mindfulness activities, paced diaphragmatic breathing, supportive counselling, cognitive behavioural therapy, biofeedback therapy, sleep hygiene, and ongoing patient education [29]. Interdisciplinary interventions should be designed in such a way that psychologists and medical care staff work together to minimize the psychological distress of patients with central sensitization pain syndromes [50].
**Pharmacological treatments**	Ensuring the availability of pharmacological treatments for CP patients at all times via the establishment of programs in conjunction with local pharmacies is a priority. Identification and implementation of strategies aimed at ensuring continuity of care provision while mitigating the risk of inadequate analgesic treatment, self-medication with potential risk of adverse events, and even treatment discontinuation, are crucial [26,51]. Prescribe pain drugs for an extended period of time—i.e., filling of prescriptions of controlled substances (opioids and psychotropics) for longer periods than usual (e.g., 60 to 90 days) [52].
**Social inequalities**	Need for strategies aimed at the reduction in disparities related to healthcare access, such as improvements in the accessibility of telehealth services (impoverished, rural, digitally illiterate populations) [41,53,54]. Need for an increased awareness, identification, and reduction in conscious and unconscious biases involved in affecting the management of CP patients from vulnerable and/or minority groups [9,55].

## Data Availability

Provided in Supplementary files.

## Supplementary Materials

The following supporting information can be downloaded at https://www.mdpi.com/article/10.3390/jcm12237233/s1, File S1. Complete search strategies used in the various electronic databases. File S2. Data extracted from the 119 articles (compiled recommendations). File S3. Summary of all the recommendations by category. Refs. [81,82] are listed in supplementary materials File S1.

## Abbreviation

Chronic pain (CP); Centre Hospitalier de l’Université de Montréal (CHUM); International Association for the Study of Pain (IASP); Fonds de développement académique du réseau (FODAR); Canadian Institutes of Health Research (CIHR); Fonds de recherche du Québec—Santé (FRQS).
