# Supplementary material for: The Calm after the Storm: A State-of-the-Art Review about Recommendations Put Forward during the COVID-19 Pandemic to Improve Chronic Pain Management
Source: J Clin Med. 2023 Nov 22;12(23):7233. doi: 10.3390/jcm12237233 (PMC10706991; doi:10.3390/jcm12237233)
Supplement: Supplementary file 1 [file jcm-12-07233-s001.zip › File S1_Search strategies.pdf]

Bibliothèque CHUM

Rapport de recherche systématique

Impact de la COVID-19 chez les personnes souffrant de douleurs chroniques

Recherche de données probantes effectuée par	Valérie Jacques, M.S.I.
Pour	Tristan Spilak
Date	1 juillet 2021

Le présent rapport est basé sur les recommandations de l'extension PRISMA-S pour rapporter les recherches documentaires en support à une revue systématique (1). La liste de contrôle complète (en anglais) est disponible à : http://www.prisma-statement.org/Extensions/Searching. Pour des recommandations complètes sur les éléments à inclure dans votre rapport de revue systématique, voir la mise à jour PRISMA 2020 (2) : http://www.prisma-statement.org/PRISMAStatement/PRISMAStatement.

La section 1 contient la méthodologie telle qu'elle devrait être rapportée dans la section du même nom de la publication.

Les sections 2 et 3 devraient être rapportées comme matériel supplémentaire dans un appendice à la publication. Des options pour la publication de ce matériel sont identifiées dans la table 3 de l'extension PRISMA-S disponible à : https://www.ncbi.nlm.nih.gov/pmc/articles/PMC7839230/table/Tab3/?report=objectonly

La section 4 contient le « PRISMA flow diagram » basé sur la version PRISMA 2020 (2).

Les résultats sont fournis séparément dans une bibliothèque EndNote.

NOTE : les éléments à inclure dans le rapport de publication sont fournis en anglais. Si besoin est, nous pouvons les fournir en français.

Table des matières

1. Méthodologie de recherche (pour l'article) 3
2. Sources et stratégies de recherche (pour l'appendice) 4
   2.1. Medline [OVID] 4
   2.2. PubMed 5
   2.3. PsycInfo [OVID] 6
   2.4. CINAHL COMPLETE [EBSCO] 8
3. Résultats par base de données et doublons supprimés 10
4. « PRISMA Flow diagram », selon la mise à jour PRISMA 2020(2) 11
5. Procédure pour obtenir les articles 12
6. Bibliographie 12

1. Méthodologie de recherche (pour l'article)

Ci-dessous, éléments tirés de PRISMA-S (1).

Information sources and methods

The following electronic databases were searched: MEDLINE (Ovid), PsycINFO (Ovid), CINAHL (EBSCOhost) and PubMed (the last 7 days).

Search strategies

Search strategies were designed by a librarian. The comprehensive literature search was initially run on 1 July 2021.The strategies were peer reviewed by another senior information specialist prior to execution using the PRESS Checklist (3).

Managing records

A total of 1 324 citations were retrieved from the databases. Duplicates were removed in EndNote by the librarian, using the method reported by W. Bramer (4).

 © Centre hospitalier de l'Université de Montréal (CHUM) 2021

2. Sources et stratégies de recherche (pour l'appendice)

2.1. Medline [OVID]

Ovid MEDLINE(R) and Epub Ahead of Print, In-Process, In-Data-Review & Other Non-Indexed Citations, Daily and Versions(R) <1946 to June 30, 2021>

#	Searches	Results
1	Chronic Pain/ or exp Neuralgia/ or exp Migraine Disorders/ or Fibromyalgia/ or exp Back Pain/ or exp Arthritis/ or exp Myofascial Pain Syndromes/	383077
2	((chronic or persistent or constant or continu* or endless or incessant or perpetual or relentless or repeat* or in for long haul or interminable or never-ending or neuropathic or myofascial or back or nerve or vertebrogenic) adj2 pain*).tw,kf,kw.	137808
3	(neuralgia* or neurodynia* or fibromyalgia* or muscular rheumatism or fibrositi* or arthriti* or polyarthriti* or lumbago or back ache* or backache* or migrain*).tw,kf,kw.	266156
4	((chronic or persistent or constant or continu* or endless or incessant or perpetual or relentless or repeat* or in for long haul or interminable or never-ending) adj2 (headache* or head pain*)).tw,kw,kf.	5331
5	1 or 2 or 3 or 4	552921
6	exp Coronavirus/	81085
7	exp Coronavirus Infections/	98955
8	COVID-19/	88400
9	(coronavirus* or corona virus* or OC43 or NL63 or 229E or HKU1 or HCoV* or ncov* or covid* or sars-cov* or sarscov* or Sars-coronavirus* or Severe Acute Respiratory Syndrome Coronavirus*).mp.	166912
10	(or/6-9) and ((20191* or 202*).dp. or 20190101:20301231.(ep).)	153934
11	10 not (SARS or SARS-CoV or MERS or MERS-CoV or Middle East respiratory syndrome or camel* or dromedar* or equine or coronary or coronal or covidence* or covidien or influenza virus or HIV or bovine or calves or TGEV or feline or porcine or BCoV or PED or PEDV or PDCoV or FIPV or FCoV or SADS-CoV or canine or CCov or zoonotic or avian influenza or H1N1 or H5N1 or H5N6 or IBV or murine corona*).mp.	56513
12	((pneumonia or covid* or coronavirus* or corona virus* or ncov* or 2019-ncov or sars*).mp. or exp pneumonia/) and Wuhan.mp.	5401
13	(2019 ncov or 2019-ncov or ncov19 or ncov-19 or 2019-novel CoV or sars coronavirus 2 or sars-cov2 or sars-cov-2 or sarscov2 or sarscov-2 or Sars-coronavirus2 or Sars-coronavirus-2 or SARS-like coronavirus* or coronavirus-19 or covid19 or covid-19 or covid 2019 or ((novel or new or nouveau) adj2 (CoV or nCoV or covid or coronavirus* or corona virus or Pandemi*2)) or ((covid or covid 19 or covid19 or covid-19) and pandemic*2) or (coronavirus* and pneumonia)).mp.	151793

14	COVID-19.rx,px,ox. or severe acute respiratory syndrome coronavirus 2.os.	4384
15	("32240632" or "32236488" or "32268021" or "32267941" or "32169616" or "32267649" or "32267499" or "32267344" or "32248853" or "32246156" or "32243118" or "32240583" or "32237674" or "32234725" or "32173381" or "32227595" or "32185863" or "32221979" or "32213260" or "32205350" or "32202721" or "32197097" or "32196032" or "32188729" or "32176889" or "32088947" or "32277065" or "32273472" or "32273444" or "32145185" or "31917786" or "32267384" or "32265186" or "32253187" or "32265567" or "32231286" or "32105468" or "32179788" or "32152361" or "32152148" or "32140676" or "32053580" or "32029604" or "32127714" or "32047315" or "32020111" or "32267950" or "32249952" or "32172715").ui.	49
16	or/12-15	151859
17	11 or 16	154461
18	17 and 20191201:20301231.(dt).	152310
19	5 and 18	1022
20	limit 19 to (yr="2019 -Current" and (english or french))	1000

2.2. PubMed

#	Searches	Results
1	(((((((chronic[Title/Abstract] OR persistent[Title/Abstract] OR constant[Title/Abstract] OR continu*[Title/Abstract] OR endless[Title/Abstract] OR incessant[Title/Abstract] OR perpetual[Title/Abstract] OR relentless[Title/Abstract] OR repeat*[Title/Abstract] OR in long haul[Title/Abstract] OR interminable[Title/Abstract] OR never-ending[Title/Abstract] OR neuropathic[Title/Abstract] OR myofascial[Title/Abstract] OR back[Title/Abstract] OR nerve[Title/Abstract] OR vertebrogenic[Title/Abstract]) AND pain*[Title/Abstract])) OR (((chronic[Other Term] OR persistent[Other Term] OR constant[Other Term] OR continu*[Other Term] OR endless[Other Term] OR incessant[Other Term] OR perpetual[Other Term] OR relentless[Other Term] OR repeat*[Other Term] OR long haul[Other Term] OR interminable[Other Term] OR never-ending[Other Term] OR neuropathic[Other Term] OR myofascial[Other Term] OR back[Other Term] OR nerve[Other Term] OR vertebrogenic[Other Term]) AND pain*[Other Term]))) OR (((neuralgia*[Title/Abstract] OR neurodynia*[Title/Abstract] OR fibromyalgia*[Title/Abstract] OR muscular rheumatism[Title/Abstract] OR fibrositi*[Title/Abstract] OR arthriti*[Title/Abstract] OR polyarthriti*[Title/Abstract] OR lumbago[Title/Abstract] OR back ache*[Title/Abstract] OR backache*[Title/Abstract] OR migrain*[Title/Abstract])) OR ((neuralgia*[Other Term] OR neurodynia*[Other Term] OR fibromyalgia*[Other Term] OR muscular rheumatism[Other Term] OR fibrositi*[Other Term] OR arthriti*[Other Term] OR polyarthriti*[Other Term] OR lumbago[Other Term] OR back ache*[Other Term] OR backache*[Other Term] OR migrain*[Other Term])))) OR ((((chronic[Title/Abstract] OR persistent[Title/Abstract] OR constant[Title/Abstract] OR continu*[Title/Abstract] OR endless[Title/Abstract]	0

OR incessant[Title/Abstract] OR perpetual[Title/Abstract] OR relentless[Title/Abstract] OR repeat*[Title/Abstract] OR long haul[Title/Abstract] OR interminable[Title/Abstract] OR never-ending[Title/Abstract]) AND (headache*[Title/Abstract] OR head pain*[Title/Abstract]))) OR (((chronic[Other Term] OR persistent[Other Term] OR constant[Other Term] OR continu*[Other Term] OR endless[Other Term] OR incessant[Other Term] OR perpetual[Other Term] OR relentless[Other Term] OR repeat*[Other Term] OR long haul[Other Term] OR interminable[Other Term] OR never-ending[Other Term]) AND (headache*[Other Term] OR head pain*[Other Term]))))) AND (((2019 ncov[Title/Abstract] OR 2019-ncov[Title/Abstract] OR ncov19[Title/Abstract] OR ncov-19[Title/Abstract] OR 2019-novel CoV[Title/Abstract] OR sars coronavirus 2[Title/Abstract] OR sars-cov2[Title/Abstract] OR sars-cov-2[Title/Abstract] OR sarscov2[Title/Abstract] OR sarscov-2[Title/Abstract] OR Sars-coronavirus2[Title/Abstract] OR Sars-coronavirus-2[Title/Abstract] OR SARS-like coronavirus*[Title/Abstract] OR coronavirus-19[Title/Abstract] OR covid19[Title/Abstract] OR covid-19[Title/Abstract] OR covid 2019[Title/Abstract] OR ((novel[Title/Abstract] OR new[Title/Abstract] OR nouveau[Title/Abstract]) AND (CoV[Title/Abstract] OR nCoV[Title/Abstract] OR covid[Title/Abstract] OR coronavirus*[Title/Abstract] OR corona virus[Title/Abstract] OR Pandemi*[Title/Abstract])) OR ((covid[Title/Abstract] OR covid 19[Title/Abstract] OR covid19[Title/Abstract] OR covid-19)[Title/Abstract] AND pandemic*[Title/Abstract]) OR (coronavirus*[Title/Abstract] AND pneumonia[Title/Abstract]))) OR ((2019 ncov[Other Term] OR 2019-ncov[Other Term] OR ncov19[Other Term] OR ncov-19[Other Term] OR 2019-novel CoV[Other Term] OR sars coronavirus 2[Other Term] OR sars-cov2[Other Term] OR sars-cov-2[Other Term] OR sarscov2[Other Term] OR sarscov-2[Other Term] OR Sars-coronavirus2[Other Term] OR Sars-coronavirus-2[Other Term] OR SARS-like coronavirus*[Other Term] OR coronavirus-19[Other Term] OR covid19[Other Term] OR covid-19[Other Term] OR covid 2019[Other Term] OR ((novel[Other Term] OR new[Other Term] OR nouveau[Other Term]) AND (CoV[Other Term] OR nCoV[Other Term] OR covid[Other Term] OR coronavirus*[Other Term] OR corona virus[Other Term] OR Pandemi*[Other Term]) OR ((covid[Other Term] OR covid 19[Other Term] OR covid19[Other Term] OR covid-19)[Other Term] AND pandemic*)[Other Term] OR (coronavirus*[Other Term] AND pneumonia[Other Term]))))) AND ((("2021/06/24"[Date - Create] : "2021/07/01"[Date - Create])))

2.3. PsycInfo [OVID]

APA PsycInfo <1806 to June Week 3 2021>

#	Searches	Results

1	Chronic Pain/ or exp Neuralgia/ or exp Migraine Headache/ or Fibromyalgia/ or exp Back Pain/ or exp Arthritis/ or exp Myofascial Pain/	32759
2	((chronic or persistent or constant or continu* or endless or incessant or perpetual or relentless or repeat* or in for long haul or interminable or never-ending or neuropathic or myofascial or back or nerve or vertebrogenic) adj2 pain*).tw,id.	30531
3	(neuralgia* or neurodynia* or fibromyalgia* or muscular rheumatism or fibrositi* or arthriti* or polyarthriti* or lumbago or back ache* or backache* or migrain*).tw,id.	22702
4	((chronic or persistent or constant or continu* or endless or incessant or perpetual or relentless or repeat* or in for long haul or interminable or never-ending) adj2 (headache* or head pain*)).tw,id.	2121
5	1 or 2 or 3 or 4	53346
6	exp Coronavirus/	2903
7	(coronavirus* or corona virus* or OC43 or NL63 or 229E or HKU1 or HCoV* or ncov* or covid* or sars-cov* or sarscov* or Sars-coronavirus* or Severe Acute Respiratory Syndrome Coronavirus*).tw,id.	6959
8	or/6-7	7193
9	8 not (SARS or SARS-CoV or MERS or MERS-CoV or Middle East respiratory syndrome or camel* or dromedar* or equine or coronary or coronal or covidence* or covidien or influenza virus or HIV or bovine or calves or TGEV or feline or porcine or BCoV or PED or PEDV or PDCoV or FIPV or FCoV or SADS-CoV or canine or CCov or zoonotic or avian influenza or H1N1 or H5N1 or H5N6 or IBV or murine corona*).tw,id.	6224
10	((pneumonia or covid* or coronavirus* or corona virus* or ncov* or 2019-ncov or sars*).tw,id. or exp pneumonia/) and Wuhan.tw,id.	143
11	(2019 ncov or 2019-ncov or ncov19 or ncov-19 or 2019-novel CoV or sars coronavirus 2 or sars-cov2 or sars-cov-2 or sarscov2 or sarscov-2 or Sars-coronavirus2 or Sars-coronavirus-2 or SARS-like coronavirus* or coronavirus-19 or covid19 or covid-19 or covid 2019 or severe acute respiratory syndrome coronavirus 2 or ((novel or new or nouveau) adj2 (CoV or nCoV or covid or coronavirus* or corona virus or Pandemi*2)) or ((covid or covid 19 or covid19 or covid-19) and pandemic*2) or (coronavirus* and pneumonia)).tw,id.	6721
12	or/9-11	6963
13	8 or 12	7217
14	5 and 13	31
15	limit 14 to (yr="2019 -Current" and (english or french))	25

2.4. CINAHL COMPLETE [EBSCO]

#	Question	Résultats
S15	S5 AND S14 Opérateurs de restriction - Date de publication: 20190101-; Langue: English, French	299
S14	S10 OR S13	54,306
S13	S11 OR S12	48,680
S12	TI ((2019 ncov OR 2019-ncov OR ncov19 OR ncov-19 OR 2019-novel CoV OR sars coronavirus 2 OR sars-cov2 OR sars-cov-2 OR sarscov2 OR sarscov-2 OR Sars-coronavirus2 OR Sars-coronavirus-2 OR SARS-like coronavirus* OR coronavirus-19 OR covid19 OR covid-19 OR covid 2019 OR severe acute respiratory syndrome coronavirus 2 OR ((novel OR new OR nouveau) N2 (CoV OR nCoV OR covid OR coronavirus* OR corona virus OR Pandemi*2)) OR ((covid OR covid 19 OR covid19 OR covid-19) and pandemic*2) OR (coronavirus* and pneumonia))) OR AB ((2019 ncov OR 2019-ncov OR ncov19 OR ncov-19 OR 2019-novel CoV OR sars coronavirus 2 OR sars-cov2 OR sars-cov-2 OR sarscov2 OR sarscov-2 OR Sars-coronavirus2 OR Sars-coronavirus-2 OR SARS-like coronavirus* OR coronavirus-19 OR covid19 OR covid-19 OR covid 2019 OR severe acute respiratory syndrome coronavirus 2 OR ((novel OR new OR nouveau) N2 (CoV OR nCoV OR covid OR coronavirus* OR corona virus OR Pandemi*2)) OR ((covid OR covid 19 OR covid19 OR covid-19) and pandemic*2) OR (coronavirus* and pneumonia)))	48,633
S11	TI (((pneumonia OR covid* OR coronavirus* OR corona virus* OR ncov* OR 2019-ncov OR sars*) AND Wuhan)) OR AB (((pneumonia OR covid* OR coronavirus* OR corona virus* OR ncov* OR 2019-ncov OR sars*) AND Wuhan)) OR (MH "pneumonia" AND TI Wuhan) OR (MH "pneumonia" AND AB Wuhan)	1,220
S10	S8 NOT S9	51,212
S9	TI ((SARS OR SARS-CoV OR MERS OR MERS-CoV OR Middle East respiratory syndrome OR camel* OR dromedar* OR equine OR coronary OR coronal OR covidence* OR covidien OR influenza virus OR HIV OR bovine OR calves OR TGEV OR feline OR porcine OR BCoV OR PED OR PEDV OR PDCoV OR FIPV OR FCoV OR SADS-CoV OR canine OR CCov OR zoonotic OR avian influenza OR H1N1 OR H5N1 OR H5N6 OR IBV OR murine corona*)) OR AB ((SARS OR SARS-CoV OR MERS OR MERS-CoV OR Middle East respiratory syndrome OR camel* OR dromedar* OR equine OR coronary OR coronal OR covidence* OR covidien OR influenza virus OR HIV OR bovine OR calves OR TGEV OR feline OR porcine OR BCoV OR PED OR PEDV OR PDCoV OR FIPV OR FCoV OR SADS-CoV OR canine OR CCov OR zoonotic OR avian influenza OR H1N1 OR H5N1 OR H5N6 OR IBV OR murine corona*))	239,645
S8	S6 OR S7	54,000
S7	TI ((coronavirus* OR corona virus* OR OC43 OR NL63 OR 229E OR HKU1 OR HCoV* OR ncov* OR covid* OR sars-cov* OR sarscov* OR Sars-coronavirus* OR Severe Acute Respiratory Syndrome Coronavirus*)) OR AB ((coronavirus* OR corona virus* OR OC43 OR NL63 OR 229E OR HKU1 OR HCoV* OR ncov* OR covid* OR sars-cov* OR sarscov* OR Sars-coronavirus* OR Severe Acute Respiratory Syndrome Coronavirus*))	50,820
S6	MH "Coronavirus"/ OR MH "COVID-19"	16,823
S5	S1 OR S2 OR S3 OR S4	157,764

S4	TI (((chronic OR persistent OR constant OR continu* OR endless OR incessant OR perpetual OR relentless OR repeat* OR in fOR long haul OR interminable OR never-ending) N2 (headache* OR head pain*))) OR AB (((chronic OR persistent OR constant OR continu* OR endless OR incessant OR perpetual OR relentless OR repeat* OR in fOR long haul OR interminable OR never-ending) N2 (headache* OR head pain*)))	2,660
S3	TI ((neuralgia* OR neurodynia* OR fibromyalgia* OR muscular rheumatism OR fibrositi* OR arthriti* OR polyarthriti* OR lumbago OR back ache* OR backache* OR migrain*)) OR AB ((neuralgia* OR neurodynia* OR fibromyalgia* OR muscular rheumatism OR fibrositi* OR arthriti* OR polyarthriti* OR lumbago OR back ache* OR backache* OR migrain*))	71,464
S2	TI (((chronic OR persistent OR constant OR continu* OR endless OR incessant OR perpetual OR relentless OR repeat* OR in fOR long haul OR interminable OR never-ending OR neuropathic OR myofascial OR back OR nerve OR vertebrogenic) N2 pain*)) OR AB (((chronic OR persistent OR constant OR continu* OR endless OR incessant OR perpetual OR relentless OR repeat* OR in fOR long haul OR interminable OR never-ending OR neuropathic OR myofascial OR back OR nerve OR vertebrogenic) N2 pain*))	69,122
S1	MH "Chronic Pain" OR MH "Neuralgia" OR MH "Migraine" OR MH "Fibromyalgia" OR MH "Back Pain" OR MH "Arthritis" OR MH "Myofascial Pain Syndromes"	68,837

3. Résultats par base de données et doublons supprimés

Exemple; la liste des bases de données variera en fonction des sources utilisées pour la RS.

Databases	Results Before Duplicate Removal	Duplicates Deleted	Results to screen After Duplicate Removal	% Retained
**Ovid Medline**	1 000	21	979	98 %
**PubMed**	0	0	0	0 %
**Ovid PsycInfo**	25	13	12	48 %
**CINAHL**	299	194	105	35 %
Totals	***1 324***	***228***	***1 096***	***83 %***

 © Centre hospitalier de l'Université de Montréal (CHUM) 2021

4. « PRISMA Flow diagram », selon la mise à jour PRISMA 2020(2)

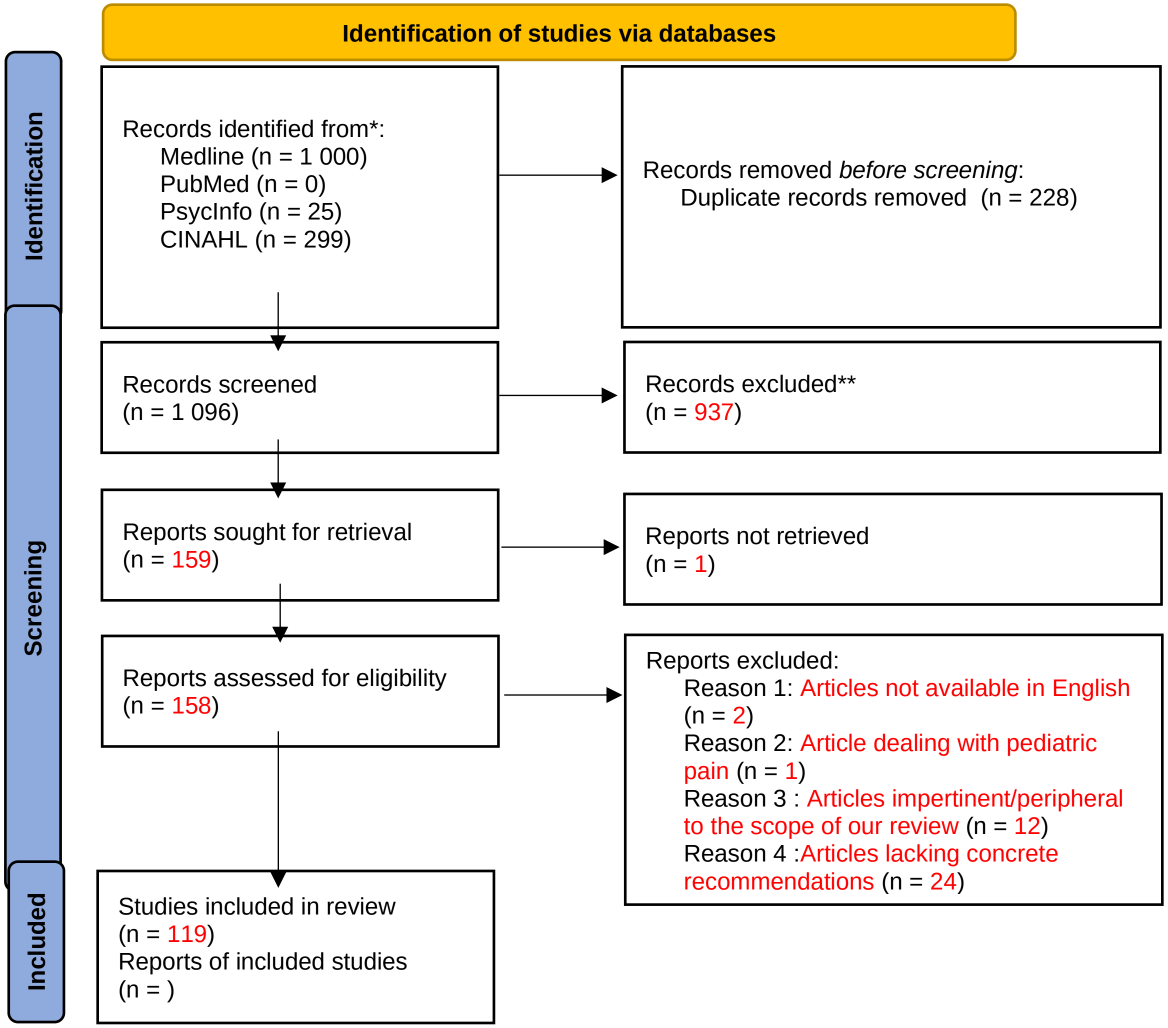

 © Centre hospitalier de l'Université de Montréal (CHUM) 2021

5. Procédure pour obtenir les articles

Lorsque vous aurez fini le tri des articles, pour obtenir le texte intégral des articles sélectionnés, voici les options :

- **Si vous êtes affilié.e. à l'Université de Montréal**, nous vous recommandons d'utiliser la fonction « Find Full Text » après avoir configuré EndNote selon les instructions disponibles à : https://bib.umontreal.ca/citer/logiciels-bibliographiques/endnote/fonctions-avancees.

- **Si vous êtes un.e employé.e du CHUM**, à partir d'un ordinateur du CHUM, nous vous recommandons d'utiliser la fonction « Find Full Text » de EndNote.

Autre option : à partir d'un ordinateur du CHUM, cliquez sur l'adresse affichée dans le champ URL pour vérifier la disponibilité d'un article soit parmi les abonnements du CHUM soit en libre accès. Si l'article est disponible, l'accès devrait être direct. Si l'article n'est pas disponible, une fenêtre s'ouvrira et vous proposera, entre autres, la possibilité de le commander.

Pour les articles dont le texte intégral n'a pas été trouvé, vous pouvez vérifier leur disponibilité en saisissant les données dans le formulaire « Rechercher une référence exacte » via la page suivante : https://gf3ey2dv2a.search.serialssolutions.com/ejp/?libHash=GF3EY2DV2A#/?language=fr-ca&titleType=JOURNALS. S'ils ne sont effectivement pas disponibles, vous aurez l'option de les commander par prêt entre bibliothèques.

Vous pouvez aussi aller directement à l'adresse suivante pour les commander en lot par prêt entre bibliothèques : https://sondage.chumontreal.qc.ca/index.php/269729?lang=fr

6. Bibliographie

1. Rethlefsen ML, Kirtley S, Waffenschmidt S, Ayala AP, Moher D, Page MJ, et al. PRISMA-S: an extension to the PRISMA Statement for Reporting Literature Searches in Systematic Reviews. Syst Rev. 2021;10(1):39. DOI: https://dx.doi.org/10.1186/s13643-020-01542-z.

2. Page MJ, McKenzie JE, Bossuyt PM, Boutron I, Hoffmann TC, Mulrow CD, et al. The PRISMA 2020 statement: An updated guideline for reporting systematic reviews. Int J Surg. 2021;88:105906. DOI: https://dx.doi.org/10.1016/j.ijsu.2021.105906.

3. McGowan J, Sampson M, Salzwedel DM, Cogo E, Foerster V, Lefebvre C. PRESS Peer Review of Electronic Search Strategies: 2015 Guideline Statement. J Clin Epidemiol. 2016;75:40-6. DOI: https://dx.doi.org/10.1016/j.jclinepi.2016.01.021.

4. Bramer WM, Giustini D, de Jonge GB, Holland L, Bekhuis T. De-duplication of database search results for systematic reviews in EndNote. J Med Libr Assoc. 2016;104(3):240-3. DOI: https://dx.doi.org/10.3163/1536-5050.104.3.014.

© Centre hospitalier de l'Université de Montréal (CHUM) 2021
